# Evaluation of postural stability based on a force plate and inertial sensor during static balance measurements

**DOI:** 10.1186/s40101-018-0187-5

**Published:** 2018-12-13

**Authors:** Chia-Hsuan Lee, Tien-Lung Sun

**Affiliations:** 1 Department of Industrial Management, National Taiwan University of Science and Technology, No. 43, Sec. 4, Keelung Road, Da’an District, Taipei, 106 Republic of China; 2 Department of Industrial Engineering and Management, Yuan Ze University, 135 Yuan Tung Road, Chungli District, Taoyuan, 320 Republic of China

**Keywords:** Static balance, Force plate, Inertial sensor, Multiscale entropy

## Abstract

**Background:**

Previous research on balance mostly focused on the assessment, training, and improvements of balance through interventions. We investigated tools commonly used to study static balance. Differences in postural stability were analyzed using multiscale entropy (MSE) and feature analysis.

**Methods:**

A force plate and inertial sensor were used to collect acceleration and center-of-pressure (COP) nonlinear signals. MSE was also used to detect fractal correlations and assess the complexity of univariate data complexity. Fifteen healthy subjects participated in the experiments. Each stood on a force plate and wore a sensor while attempting to maintain postural stability for 30 s in four randomized experiments to evaluate their static balance via a copositive experiment with eyes open/closed and with standing on one foot or both feet. A Wilcoxon-signed rank test was used to confirm that the conditions were significant. Considering the effect of the assessment tools, the influence of the visual and lower limb systems on postural stability was assessed and the results from the inertial sensor and force plate experiments were compared.

**Results:**

Force plate usage provided more accurate readings when completing static balance tasks based on the visual system, whereas an inertial sensor was preferred for lower-limb tasks. Further, the eyes-open-standing-on-one-foot case involved the highest complexity at the *X*, *Y*, and *Z* axes for acceleration and at the ML axis for COP compared with other conditions, from which the axial directions can be identified.

**Conclusions:**

The findings suggested investigation of different evaluation tool choices that can be easily adapted to suit different needs. The results for the complexity index and traditional balance indicators were comparable in their implications on different conditions. We used MSE to determine the equipment that measures the postural stability performance. We attempted to generalize the applications of complexity index to tasks and training characteristics and explore different tools to obtain different results.

**Trial registration:**

This study was approved by the Research Ethics Committee of National Taiwan University and classified as expedited on August 24, 2017. The committee is organized under and operates in accordance with Social and Behavioral Research Ethical Principles and Regulations of National Taiwan University and government laws and regulations.

## Background

Balance affects the quality of life and plays a significant role in fall risk. Current research on balance mostly has focused on the assessment, training, and improvements through interventions. Clinically, falling is the unexpected change in body position due to the body’s center of gravity being out of balance [1]. In clinical settings, the most commonly used tests to examine a subject’s fall risk in determining whether the balance ability is present and include Timed Up and Go test in which the measure of function correlates to balance and fall risk and unipedal stance test with eyes open/closed which is a method of quantifying static balance ability [2]. In contrast, laboratory research involves subjects to execute specific movements and uses different parameters to observe their limb movements and physical responses for examining how they maintain balance. Examples include performing tasks under different visual setting [3, 4], standing on different materials [5], standing still, walking, and performing other tasks [6] as well as comparing the balancing ability between different populations [3, 7]. Balance training has also been performed using different rehabilitation therapy systems such as cycling [8] and partial body weight support on a treadmill [9]. These studies mostly use motion capture systems and force plates for real-time acquisition of subject limb movements to evaluate balance. Moe-Nilssen et al. [10] also employed a three-dimensional accelerometer worn on the subject’s lower back, near the center of gravity. Wearable accelerometers are a viable technology for assessing fall risk and have joined clinical and laboratory methods as acceptable tools [11]. Wearable accelerometers measure changes in the acceleration of the triaxial axis while maintaining a certain position. If these changes have a larger amplitude, the measurement value of accelerometers will also be larger. Previous studies have used sensors to investigate fall prevention, assess falls [12–14] and conduct daily monitoring [15]. Although one factor known to contribute to falls is balance ability [11, 16], few studies have considered balance evaluation tools rather than or in addition to performance.

The evaluation tool used affects the evaluation results [17]. Most studies using static balance have employed force plates [6, 18–25], which have been shown to effectively assess the center-of-pressure (COP) and thus the balance performance [16]. For example, the elderly often have reduced strength in the lower limb, resulting in increased COP displacement and indicating low balance and muscle weakness’ contribution to postural instability and falls [5]. The displacement trajectory caused by standing balance has typically been studied using the COP displacement of the body’s swing. However, it is difficult to collect time series data to account for the swing around the coordinate pair (*X*, *Y*), where the *X*-axis is the time series and the *Y*-axis is the amplitude intensity. When the amplitude intensity is the only information obtained, the signal’s meaning cannot be explained. Therefore, the front-to-back and left-to-right trajectories are determined using a separate force plate. The COP data are related to force (*F*) and moment (*M*), including *Fx*, *Fy*, *Fz*, *Mx*, *My*, and *Mz*; thus, both force and moment are used to evaluate postural stability.

Advances in sensor and data acquisition technologies have made it possible to record real-world signals containing multiple data channels in a coherent way, even with large dynamic differences between channels [26]. COP and acceleration are nonlinear, objectively collected physiological signals of attitude stability; therefore, no conclusion can be drawn if these signals are plainly presented. Signal complexity or regularity is represented by a quantized value, known as complexity [10], to distinguish differences between nonlinear data. Established complexity measures typically operate a single scale and thus fail to quantify inherent long-range correlations in real-world data, which is a key feature of complex systems. The recently introduced multiscale entropy (MSE) method can detect fractal correlations and has successfully been used to assess the complexity of univariate data [26]. Because of the instability of wearable accelerometer triaxial signal measurements [27], empirically collected COP data physiological signals are nonlinear [17]. Complexity can be easily appreciated when faced in practice settings [28] and complex systems are neither absolutely regular nor absolutely random [29, 30]. Thus, the MSE method [31, 32] was proposed to measure the complexity of finite-length time series. Previous studies have employed this method to analyze COP to investigate postural stability [19, 20]. Acceleration sensors have also been used to collect acceleration information and investigate postural stability [33–38]. Mayagoitia et al. [33] also investigated sensor and force plate features to determine the correlation; however, no published studies have examined whether entropy measurements respond differently to postural stability in different equipment and which entropy measurement show more sensitive response to stimuli.

Therefore, an experiment was designed to examine the features because MSE has been studied with both sensors and force plates. MSE is also investigated as a shared function to explore the correlation between the two and the usability of sensors measuring postural stability. Herein, we not only attempted to discuss that the discernibility of MSE is further confirmed to understand the versatility of the sensor and force plate but also discussed the evaluation tools during static balance measurements.

## Methods

While maintaining postural stability depends on the vestibular system, proprioception receptors, and visual system, studies evaluating these three systems individually in balance tests have confirmed that the visual system is particularly crucial in influencing balance [34]. Cha et al. showed that subjects’ center of gravity moved more when their eyes were closed than when they were open [35], whereas Rose [36] found a much larger center of gravity displacement when subjects stood one-legged with their eyes closed in comparison with that obtained in a stance with the eyes open. Static balance studies have also already shown comparable results. Thus, four previously used conditions to maintain postural stability were used to study balance tool feasibility: eyes open, standing on both feet (OB); eyes closed, standing on both feet (CB); eyes open, standing on one foot (OO); and eyes closed, standing on one foot (CO). These conditions were randomly assigned to subjects. During data analysis, MSE and features were used to calculate balance indicators.

### Subjects

Fifteen healthy individuals, 13 male and 2 females, between the ages of 21 and 25 (age 22.6 ± 1.55 years, height 173.2 ± 8.86 cm, weight 68.67 ± 14.22 kg) participated in this study to obtain a standard for evaluating postural stability. None of the participants had central nervous system medical issues or relevant skeletal or muscular diseases.

### Instrument information

#### AMTI multi-axis force plates

**Fig. 1:**
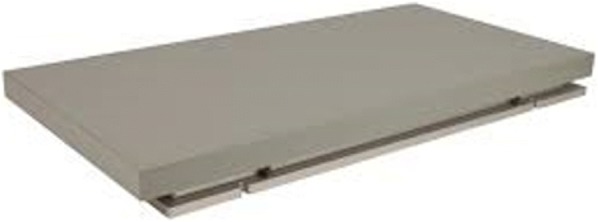
Six-axis AMTI force plate

A six-axis AMTI force plate (AMTI OR6-7-2000; Advanced Mechanical Technology, 2010; length 50.8 cm; width 46.4 cm, height 8.3 cm; weight 28.18 kg) was used to collect COP data (Fig. 1). AMTI OR6-7-2000 is constructed from aluminum and can collect *F* and *M* data on three axes each: *F*_*x*_, *F*_*y*_, *F*_*z*_, *M*_*x*_, *M*_*y*_, and *M*_*z*_. The signal amplifier amplifies the collected data for ease of analysis before sending it to to a computer with AMTINerForce software for analysis. A data collection sampling frequency of 100 Hz and a measurement time of 30 s were used for each action. COP movement track data (in mm) were collected for each subject and decomposed into mediolateral (ML) and anterior-posterior (AP) components for analysis.

#### Inertial sensor

**Fig. 2:**
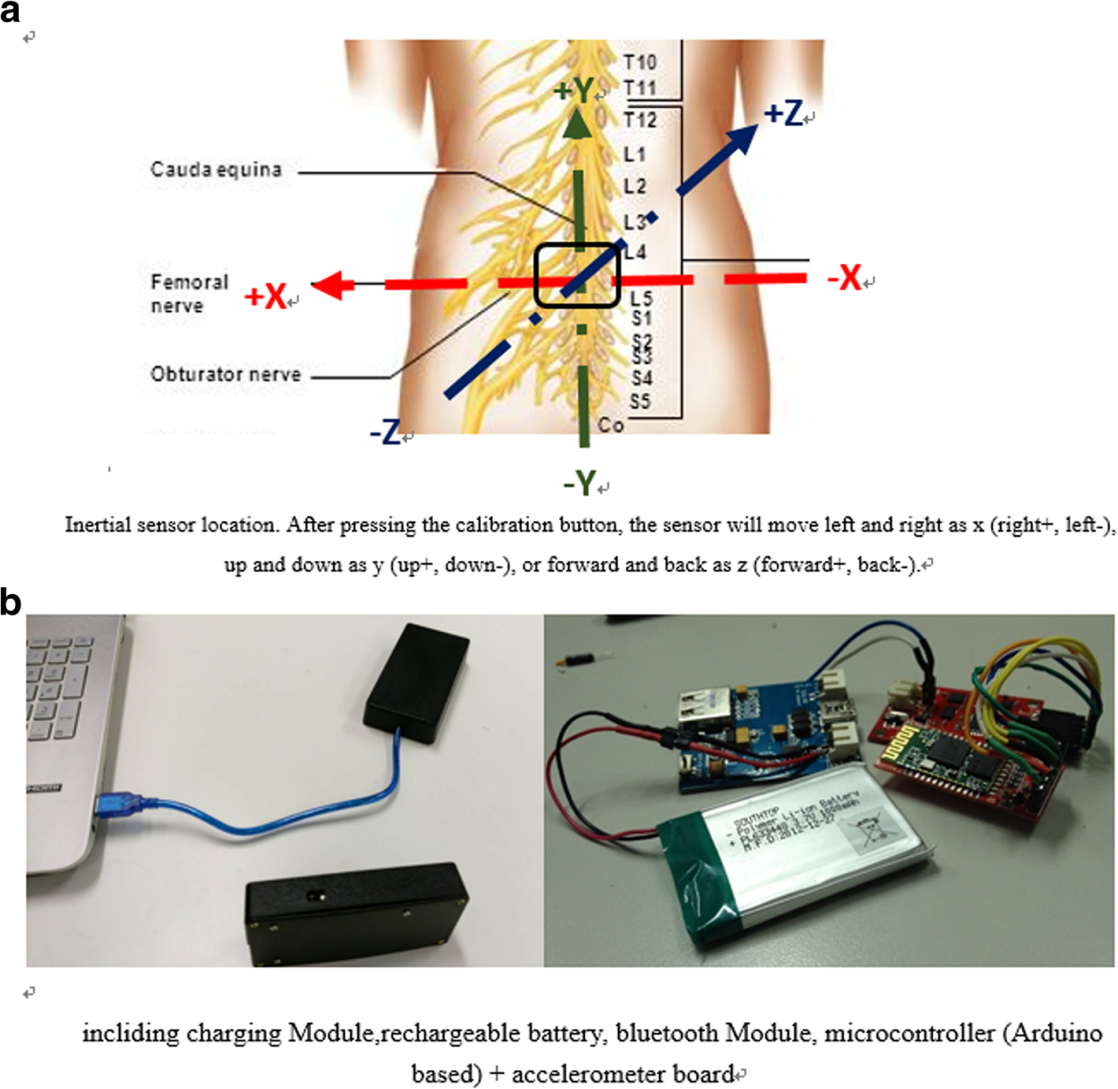
Inertial sensor. **a** Inertial sensor location. After pressing the calibration button, the sensor will move left and right as *x* (right+, left-), up and down as *y* (up+, down-), or forward and back as *z* (forward+, back-). **b** Wireless tri-axial accelerometer system (Freescale RD3152MMA7260Q)

The inertial sensor has a built-in microelectromechanical system accelerometer, gyroscope, and magnetometer and can thus collect acceleration and rotation angle data. Microelectromechanical system accelerometer sensors can be distinguished using four sensing classifications based on different power transfers: piezoelectric, piezoresistive, electrostatic, and capacitive. A capacitive sensor was used herein. The accelerometer was placed on the subject’s lower back, covering the pelvis, sacrum, and L3 to L5 vertebrae, as shown in Fig. 2. This is the most common sensor location and was the only location used in 65% of studies [23]. Changes in external forces cause movement, resulting in voltage or current changes that produce a vibration signal. Accelerometers typically use object displacement, which is then converted to a digital signal for processing. The most common detection method for accelerometers in the microelectromechanical system is capacitance. This detection method offers high precision, high stability, low power consumption, a simple structure, and lack of susceptibility to noise or temperature fluctuations. The sampling bandwidth of this device is for detecting human motion. The magnetometer measures the size and direction of magnetic fields near the device. Herein, the magnetometer’s direction function was used as each subject wore a wireless triaxial accelerometer system (Freescale RD3152MMA7260Q, Freescale Semiconductor-NXP, Austin, TX, USA) on a belt around the waist when testing postural stability. A battery, power switch, and wireless board were installed on a rigid circuit board glued to the back of the belt, as shown in Fig. 2.

### Protocols

**Fig. 3:**
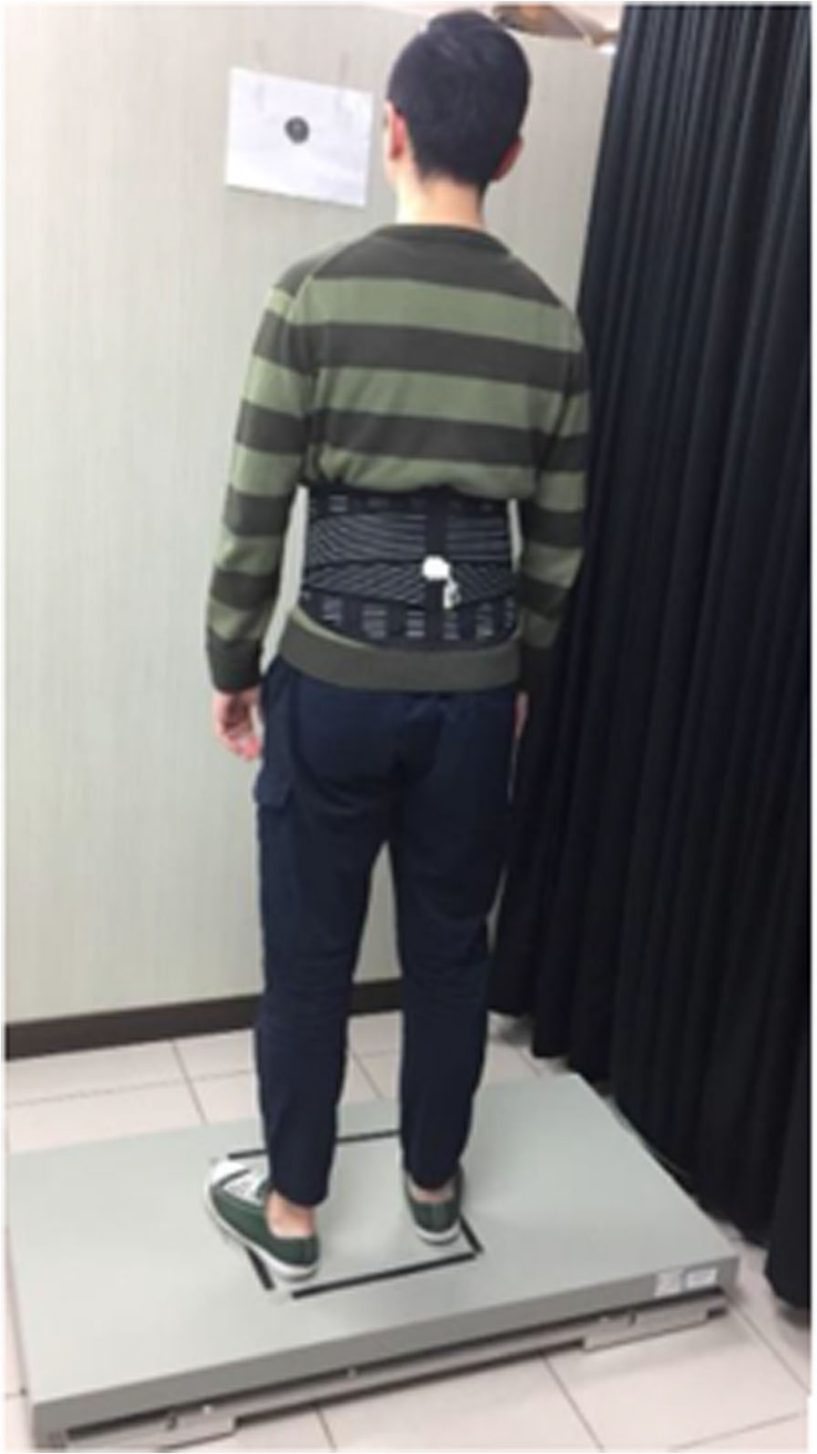
A subject standing on the plate and wearing the sensor

Inertial sensors were placed on the 15 adult subjects standing on a force plate to collect COP sway and acceleration data in order to determine whether results from the inertial sensor were similar to those from the force plate. MSE analysis was used for the data analysis of the index and features of both machines. Visual system tests were developed and conducted with opened or closed eyes. To minimize the influence from the environment during the open eye test, a black focal spot was placed in front of the test subject as shown in Fig. 3. Data collection was also divided into single- and double-legged stance categories. Standing on one leg is a posture used in daily life and employed when navigating stairs, stepping over obstacles, and walking normally. People are most likely to fall due to a shift in the center of gravity while standing on one leg. Previous studies have used one-legged stance as a posture for balance training [17, 37–39]. Four experimental conditions were used: OB, CB, OO, and CO.

The four conditions were randomized for each participant, and the experimental process comprised of four parts. First, participants completed the test subject consent form and provided basic information. The interviewer then adjusted the sensor belt, determined the foot the subject used to kick, and equipped the subject with the sensor belt (see Fig. 3) while the subject stood on the force plate before performing the test. After ensuring that the data were saved, the interviewer removed the sensor belt, which marked the completion of the experiment. The data were then analyzed via a statistical approach that included descriptive statistics such as mean and standard deviation and a Wilcoxon signed-rank test to evaluate the observed differences.

### Multiscale entropy (MSE)

Costa et al. introduced the MSE method, which performs multiple coarse-graining operations on data (thus defining temporal scales) and calculates sample entropy for each defined scale [31, 32]. The MSE method quantifies the signal complexity that remains hidden in standard methods where the temporal scales of a signal are not processed separately [26]. MSE analysis can be divided into three stages: coarse graining, sample entropy, and complexity.

During coarse graining, time series are divided into multiple specifications for a variety of time segments and spatial specifications to calculate entropy using Eq. (1), with *y* being the data point, *τ* being the scale of segmentation, and *N* being the size of the original dataset.

1 $$ {y_i}^{(x)}=\frac{1}{\tau }{\sum}_{i-\left(j-1\right)x+1}^{jx}{f}_i,1\le j\le \frac{N}{\tau }. $$

This adds an additional time scale which is added to the time series while calculating each time series. MSE calculation is based on sample entropy (SampEn), which is a single-scale analysis. MSE involves analysis using multiple scales. Thus, SampEn is necessarily calculated via a six-step process:

Step 1: The embedding dimension (*m*), used to understand repeatability and regularity of data in a time series, and tolerance (*r*), a constant, are set. It is typically recommended that m be set to 2 or 3 [40], and Pincus [41] recommends setting an r value between 0.1 and 0.2.

**Fig. 4:**
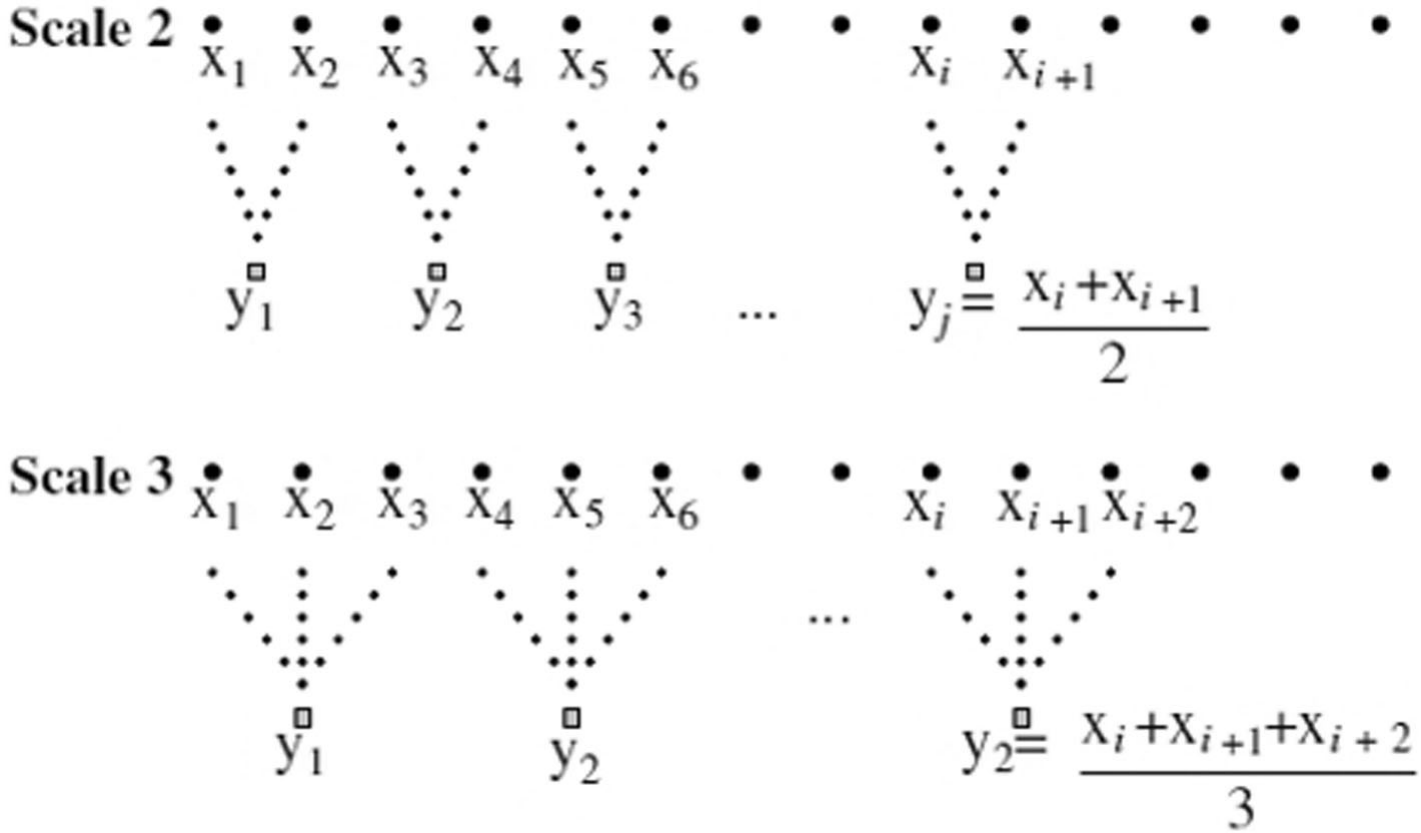
Step two, schematic of multi-scale entropy [31]

Step 2: Herein, m was set to 2 as a time series data reference. For example, for time series *X* = (*x*_1_,…,*x*_7_), when *m* = 2, the comparison unit of the group becomes {(*x*_1_, *x*_2_), (*x*_2_, *x*_3_),…,(*x*_6_, *x*_7_)}. Here, the comparison begins with the first group (*x*_1_, *x*_2_), which is then compared with the other groups, as shown in Fig. 4.

Step 3: The maximum value of the distances between the first group and remaining groups are calculated via Eq. (2):

2 $$ d\left[{x}_{i,}\ {x}_j\right]=\max \left[\left|{x}_{i+k-1}-{x}_{j+k-1}\right|\right],1\le k\le m,i\le N-m,j\le N-m. $$

Step 4: Here, the results from Eq. (2) and r*S are used as a comparison method, where *S* is the original time series on which the alignment is based. If *d*[[*x*_*i*_*x*_*j*_] is smaller than r*S, then the two compared groups can be considered similar. Therefore, similar numbers *n*_*i*(*m*)_ plus 1 are accumulated and the probability of a similar number (*C*_*i(m)*_) can be calculated using Eq. (3):

3 $$ {\mathrm{C}}_{i(m)}=\frac{n_{i(m)}}{N-m},1\le i\le N-m. $$

Step 5: Here, step 1 is repeated after changing the original data from m to m + 1. Steps 2 and 3 were also repeated; the cumulative similarity *n*_*i(m)*_ and probability of occurrence *C*_*i(m)*_ thus increase as shown in Eq. (4):

4 $$ {\mathrm{C}}_{i(m)}=\frac{n_{i\left(m+1\right)}}{N-m-1},1\le i\le N-m-1. $$

Step 6: Sample entropy can then be calculated by taking the negative natural logarithm of the average *C*_*i(m + 1)*_ value over the average *C*_*i(m)*_ value:

5 $$ \mathrm{Sample}\ \mathrm{Entropy}\left(m,r,N\right)=-\ln \left(\frac{\sum {\mathrm{C}}_{\frac{i\left(m+1\right)}{N-m-1}}}{\sum \frac{{\mathrm{C}}_{i(m)}}{N-m}}\right). $$

**Fig. 5:**
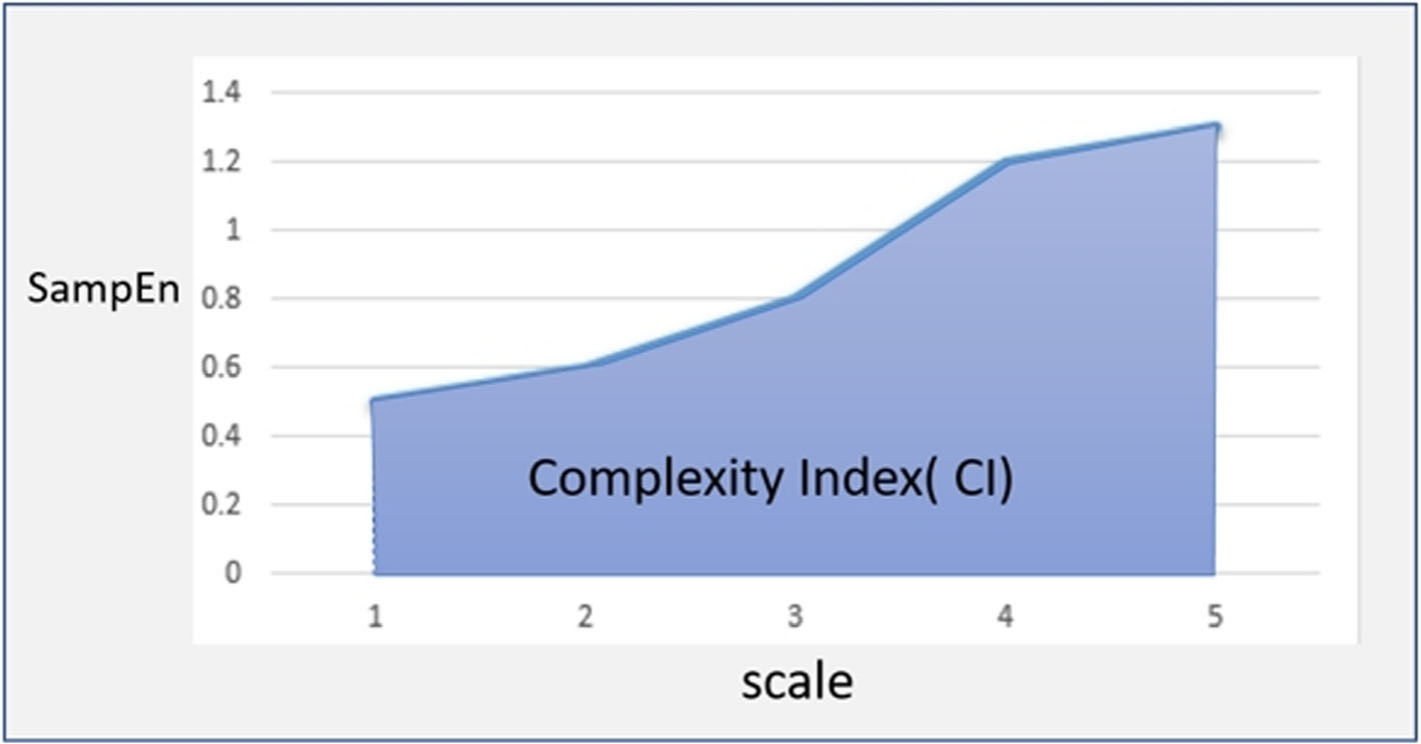
Complexity index

Finally, the complexity index (CI) can be calculated by Eq. (6) because the sample entropy is used as a scale factor function to calculate the area under the CI, as shown in Fig. 5.

6 $$ \mathrm{CI}={\sum}_{i=1}^N\mathrm{Sample}\ \mathrm{Entropy}(i). $$

### Features of force plate COP

The sampling frequency and collection time affects the number of data points collected. Additionally, each participant may stand at a different position on the force plate during measurement. Therefore, each data point should first be zeroed before follow-up formulas are calculated for the COP index analysis. Average values were first calculated for the left-to-right and front-to-back directions, as shown in Eqs. (7) and (8), respectively, where AP_0_ and ML_0_ are original forward–backward and left–right data of the pressure midpoint, respectively.

7 $$ \overline{\mathrm{AP}}=\frac{1}{N}{\sum}_{n=1}^N{\mathrm{AP}}_O\left[n\right] $$

8 $$ \overline{\mathrm{ML}}=\frac{1}{N}{\sum}_{n=1}^N{\mathrm{ML}}_O\left[n\right] $$

The original data points and average values were then subtracted to complete coordinate zeroing, as shown in Eqs. (9) and (10), where *N* is the total data length:

9 $$ \mathrm{AP}\left[n\right]={\mathrm{AP}}_O\left[n\right]-\overline{\mathrm{AP}}\ n=1,\dots N, $$

10 $$ \mathrm{ML}\left[n\right]={\mathrm{ML}}_O\left[n\right]-\overline{\mathrm{ML}}\ n=1,\dots N. $$

The measurement indexes were then calculated using force plate data as follows:

1. Total excursions (TOTEX) are calculated as the respective distance between the AP and ML directions of the COP from the origin:

11 $$ \mathrm{TOTEX}={\sum}_{n=1}^{N-1}\sqrt{{\left(\mathrm{AP}\left[n+1\right]-\mathrm{AP}\left[n\right]\right)}^2+{\left(\mathrm{ML}\left[n+1\right]-\mathrm{ML}\left[n\right]\right)}^2}. $$

2. Total excursions-AP (TOTEX_AP_) are calculated as the distance between the COP-AP direction and the origin:

12 $$ {\mathrm{TOTEX}}_{\mathrm{AP}}=\sqrt{{\left(\mathrm{AP}\left[n+1\right]-\mathrm{AP}\left[n\right]\right)}^2}. $$

3. Total excursions-ML(TOTEX_ML_) are calculated as the distance between the COP-ML direction and the origin:

13 $$ {\mathrm{TOTEX}}_{\mathrm{ML}}=\sqrt{{\left(\mathrm{ML}\left[n+1\right]-\mathrm{ML}\left[n\right]\right)}^2}. $$

4. Mean distance (MDIST) is calculated as the distance between the COP-AP and ML directions from the origin, where N is the total length of the data:

14 $$ \mathrm{RD}\left[\mathrm{n}\right]=\sqrt{{\mathrm{AP}}_n^2+{\mathrm{ML}}_n^2},n=1,\dots, N. $$

5. Mean distance-AP (MDIST_AP_) is calculated as the average of the absolute value of the COP-AP direction:

15 $$ {\mathrm{MDIST}}_{\mathrm{AP}}=\frac{1}{N}{\sum \limits}_{n=1}^N\mid \mathrm{AP}\left[n\right]\mid . $$

6. Mean distance-ML(MDIST_ML_) is calculated as the average of the absolute value of the COP-ML direction:

16 $$ {\mathrm{MDIST}}_{\mathrm{ML}}=\frac{1}{N}{\sum \limits}_{n=1}^N\mid \mathrm{ML}\left[n\right]\mid . $$

7. Mean velocity (MVELO) is calculated as TOTEX over total time *T*:

17 $$ \mathrm{MVELO}=\frac{TOTEX}{T}. $$

8. Mean velocity-AP (MVELO_AP_) is calculated as TOTEX in the AP direction over total time *T*:

18 $$ {\mathrm{MVELO}}_{\mathrm{AP}}=\frac{{\mathrm{TOTEX}}_{\mathrm{AP}}}{T}. $$

9. Mean velocity-ML (MVELO_ML_) is calculated as TOTEX in the ML direction over total time *T*:

19 $$ {\mathrm{MVELO}}_{\mathrm{ML}}=\frac{{\mathrm{TOTEX}}_{\mathrm{ML}}}{T}. $$

10. Root-mean-square distance (RDIST) is calculated as the square root of the sum of the squared RD:

20 $$ \mathrm{RDIST}=\sqrt{\frac{1}{N}{\sum}_{n=1}^N\mathrm{RD}{\left[n\right]}^2}. $$

11. Root-mean-square distance-AP (RDIST_AP_) is calculated as the square root of the average value of the squared AP signal:

21 $$ {\mathrm{RDIST}}_{\mathrm{AP}}=\sqrt{\frac{1}{N}{\sum \limits}_{n=1}^N\mathrm{AP}{\left[n\right]}^2}. $$

12. Root-mean-square distance-ML (RDIST_ML_) is calculated as the square root of the average squared ML signal:

22 $$ {\mathrm{RDIST}}_{\mathrm{ML}}=\sqrt{\frac{1}{N}{\sum \limits}_{n=1}^N\mathrm{ML}{\left[n\right]}^2}. $$

13. The 95% confidence circle area (95% CC AREA) was then obtained. First, *Z*_0.95_ = 1.645 was obtained using a normal distribution. The calculated square root *S*_*RD*_ can then be found by subtracting the average square distance from the squared quadratic mean distance:

23 $$ {S}_{\mathrm{RD}}=\sqrt{{\mathrm{RD}\mathrm{IST}}^2-{\mathrm{MDIST}}^2}. $$

The 95% confidence level can then be calculated by squaring the sum of the product of the mean distance and the product of *Z*_0.95_ and *S*_*RD*_ multiplied by π:

24 $$ 95\%\mathrm{CC}\ \mathrm{AREA}=\pi {\left(\mathrm{MDIST}+{Z}_{0.95}\ast {S}_{\mathrm{RD}}\right)}^2. $$

### Inertial acceleration sensor features

The sensor was capable of reading accelerations on three different axes: *X*, *Y*, and *Z*. Readings of a file belonging to a test subject who completed the test in an average time of 30 s are obtained as follows.

## Mean absolute linear acceleration (MALA) is calculated using an equation proposed by Capela et al. [42]

25 $$ \mathrm{MALA}.={\sum}_{i=1}^N\frac{\left|{S}_i\right|}{N}={\sum}_{i=1}^N\frac{\left|{S}_i\right|}{N},S=\mathrm{sequence},N=\mathrm{total}\ \mathrm{number}\ \mathrm{of}\ \mathrm{units} $$

where *S* is the sequence and *N* is the total number of units. As sensor-measured acceleration is directional, adding the average arithmetic mean to the absolute value can yield one of the most direct indicators of acceleration magnitude.

## Root mean square (RMS), also known as the square average, expresses the generalized mean of the quadratic and is often used as the average of signals. RMS was calculated using an equation proposed by Chen [43]

26 $$ \mathrm{RMS}=\sqrt{\frac{\sum_{i=1}^N{S}_i}{N}},S=\mathrm{sequence},N=\mathrm{total}\ \mathrm{number}\ \mathrm{of}\ \mathrm{units},i=1,2,\dots \dots, N. $$

1. Mean absolute deviation (MAD) is often used as a discrete property for understanding sequences and was calculated using an equation proposed by Chen [43]

27 $$ \mathrm{MAD}=\frac{\sum_{i=1}^N\mid {S}_i-\overline{S}\mid }{N},S=\mathrm{sequence},N=\mathrm{total}\ \mathrm{number}\ \mathrm{of}\ \mathrm{units}. $$

2. The simple moving average of mean of range (SMA of Range) is used to minimize the influence of outliers. The original method of taking the full range of information between the minimum and maximum values from the entire series is prone to calculate too many outliers, resulting in insufficiently accurate results. Therefore, adding a simple moving average method while calculating a window parameter’s full range reforms the original data. The resulting calculated value changes accordingly with different window parameters; a larger window indicates more data points that represent a sample. In our study, when the window parameter = 30, it begins to flatten because the sensors can collect 30 data points per second. The SMA of Range was calculated with an equation proposed by Capela [42], where *W* indicates the window parameter.

28 $$ \mathrm{SMA}\ \mathrm{of}\ \mathrm{Range}=\frac{\sum_{i=1}^{N-\left(W-1\right)}{\mathrm{SMA}}_{i,W}}{N-\left(W-1\right)} $$

$$ \mathrm{where}\ {\mathrm{SMA}}_{i,W}=\frac{\mathrm{Range}\left({S}_i,\dots \dots, {S}_{i+W-1}\right)}{W},W=\mathrm{Window},N=\mathrm{total}\ \mathrm{number}\ \mathrm{of}\ \mathrm{units} $$

3. The simple moving average of mean of variance (SMA of Variance) is used to determine the average amount of variation in each window. Variance indicates the discreteness of a set of numerical values, reflecting the degree of dispersion among individuals in a group and adding a simple moving average. The SMA of Variance varies with varying window parameters as larger window parameters indicate a greater number of data points representing a sample. In this study, when we set the window parameter to 30, the value flattens significantly because the test subject’s wristband collects 30 data points per second. The SMA of Variance was calculated using an equation by Capela [42].

29 $$ \mathrm{SMA}\ \mathrm{of}\ \mathrm{Variance}=\frac{\sum_{i=1}^{N-\left(W-1\right)}{\mathrm{SMA}}_{i,W}}{N-\left(W-1\right)}, $$

$$ \mathrm{where}\ {\mathrm{SMA}}_{i,W}=\frac{\sum_{i=1}^N{\left({S}_i-\overline{S}\right)}^2}{W-1},S=\mathrm{sequence},W=\mathrm{Window},N=\mathrm{total}\ \mathrm{number}\ \mathrm{of}\ \mathrm{units}. $$

4. The zero cross rate (ZCR)is the number of lines between two points that pass through zero acceleration which indicates a change in the direction of force. ZCR is used to determine the percentage change of axis acceleration to the total number of points and was calculated using an equation by Chen [43]:

30 $$ \frac{1}{N-1}{\sum \limits}_{i=1}^{N-1}{1}_{R<0}\left({S}_i{S}_{i-1}<0\right),S=\mathrm{sequence},N=\mathrm{total}\ \mathrm{number}\ \mathrm{of}\ \mathrm{units}. $$

5. The correlation between axes (CBA), specifically between the *X*, *Y*, and *Z* axes, is represented by *ρ*_(*S*_1_*S*_2_), with *S*_1_ and *S*_2_ representing any two of the three axes. The CBA is calculated using Eq. (31), originally from Capela [42], and the following conditions:
1) There are positive relative and negative relative correlations.

$$ \left|{\rho}_{S_1{S}_2}\right|\le 1 $$

2) When $\left|{\rho}_{S_1{S}_2}\right|=1$, A and B are completely related and there is a linear function between them. When $\left|{\rho}_{S_1{S}_2}\right|>0.8$, A and B are highly related. When $0.3\le \left|{\rho}_{S_1{S}_2}\right|\le 0.8$, A and B are moderately related. When $\left|{\rho}_{S_1{S}_2}\right|<0.3$, A and B are less related to little or no degree.

31 $$ {\rho}_{AB}=\frac{\mathrm{Cov}\left({S}_1,{S}_2\right)}{\sqrt{D\left({S}_1\right)}\sqrt{D\left({S}_2\right)}} $$

$$ \mathrm{where}\ \mathrm{Cov}\left({S}_1,{S}_2\right)=\frac{\sum_{i=1}^N\left({S_1}_i-{\mu}_{S_1}\right)\times \left({S_2}_i-{\mu}_{S_2}\right)}{N-1},S=\mathrm{sequence},N=\mathrm{total}\ \mathrm{number}\ \mathrm{of}\ \mathrm{units}. $$

## Results

The analysis and discussion is divided into three main parts. First, the force plate and sensor results were calculated. Then, a Wilcoxon signed-rank test to confirm that the conditions are significant, i.e., the sample size large enough to test statistical assumptions. The MSE method was then used to analyze force plate and acceleration sensor data. Finally, the results from the force plate and acceleration sensor were compared with the MSE results and used a Wilcoxon signed-rank test used to verify the four conditions.

### Results from the force plate COP feature

**Fig. 6:**
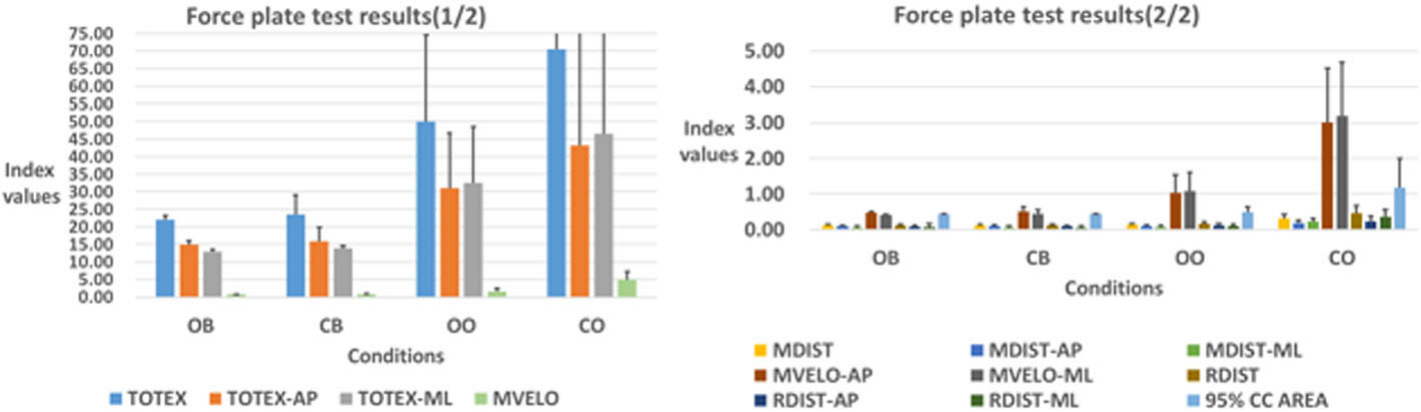
Force plate test results showing a comparison between figures (due to large differences, overly low values were compressed as they could not be taken into account easily. The figure is divided into two parts for easier judgment of values)

The data collected from the four static balance measurements performed using the force plate are presented in Table 1 and Fig. 6. All the COP features are better in smaller values. A simple observation of score indexes indicated that OB values were lower for all actions, indicating the most stable overall performance, as expected. The visual system and single- vs. double-legged stance appeared to have both influenced subjects’ balance. Initial results agree well with the results of previous studies; thus, different equipment can be used for conducting the same experiment.

**Table 1:** Force plate test results

	OB	CB	OO	CO
TOTEX	22.04 ± 1.19	23.5 ± 5.62	50.05 ± 24.46	70.53 ± 67.4
TOTEX-AP	14.93 ± 1.09	15.9 ± 3.94	31.12 ± 15.69	43.2 ± 42.06
TOTEX-ML	12.95 ± 0.64	13.91 ± 0.64	32.54 ± 16.04	46.52 ± 44.7
MDIST	0.13 ± 0.03	0.13 ± 0.03	0.14 ± 0.03	0.32 ± 0.11
MDIST-AP	0.10 ± 0.03	0.10 ± 0.04	0.10 ± 0.04	0.18 ± 0.09
MDIST-ML	0.06 ± 0.05	0.06 ± 0.05	0.08 ± 0.04	0.23 ± 0.09
MVELO	0.73 ± 0.03	0.77 ± 0.18	1.59 ± 0.83	4.91 ± 2.29
MVELO-AP	0.49 ± 0.03	0.52 ± 0.13	1.03 ± 0.52	3.02 ± 1.5
MVELO-ML	0.42 ± 0.02	0.45 ± 0.11	1.08 ± 0.53	3.19 ± 1.5
RDIST	0.12 ± 0.03	0.13 ± 0.03	0.17 ± 0.05	0.46 ± 0.22
RDIST-AP	0.09 ± 0.03	0.1 ± 0.03	0.12 ± 0.05	0.24 ± 0.14
RDIST-ML	0.08 ± 0.09	0.06 ± 0.05	0.10 ± 0.05	0.36 ± 0.20
95% CC AREA	0.442 ± 0.00	0.443 ± 0.00	0.50 ± 0.145	1.18 ± 0.82

Unit: mm

A Wilcoxon signed-rank test was used to compare the results of the actions to explore the uses of the equipment, as summarized in Table 2. Judging from each index’s discrimination, MDIST, MDIST-AP, RDIST, RDIST-AP, and 95% CC AREA were found to distinguish the results of various static balance measurement operations.

**Table 2:** *p* value of COP metrics for the static posture

	OB–CB	OB–OO	OB–CO	CB–OO	CB–CO	OO–CO
TOTEX	0.57	0.001*	0.002*	0.001*	0.005*	0.478
TOTEX-AP	0.594	0.001*	0.006*	0.001*	0.011*	0.496
TOTEX-ML	0.345	0.001*	0.001*	0.001*	0.002*	0.46
MDIST	0.005*	0.003*	0.001*	0.012*	0.001*	0.001*
MDIST-AP	0.004*	0.001*	0.001*	0.001*	0.001*	0.001*
MDIST-ML	0.053	0.233	0.001*	0.496	0.001*	0.001*
MVELO	0.591	0.001*	0.001*	0.001*	0.001*	0.001*
MVELO-AP	0.695	0.001*	0.001*	0.001*	0.001*	0.001*
MVELO-ML	0.393	0.001*	0.001*	0.001*	0.001*	0.001*
RDIST	0.025*	0.001*	0.001*	0.001*	0.001*	0.001*
RDIST-AP	0.046*	0.002*	0.001*	0.002*	0.001*	0.004*
RDIST-ML	0.151	0.01*	0.001*	0.001*	0.001*	0.001*
95% CC AREA	0.002*	0.001*	0.001*	0.001*	0.001*	0.001*

*Significance, *p* < 0.05

### Results from the inertial sensor for acceleration feature

**Fig. 7:**
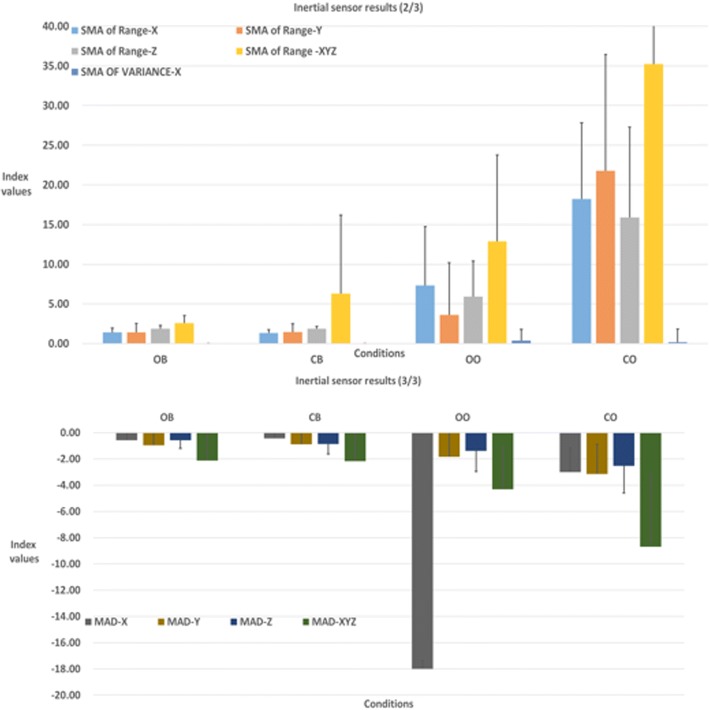
Inertial sensor results (due to the value calculated by different indexes, the range size is too large, so the graph is divided into two parts)

A cursory observation of the index scores of accelerations showed that the smaller values were better, and most situations of the feature index showed that CB performed the best for most actions of the indexes, which does not agree with expected balance results due to the visual system and ontological sensory receptors in humans (Fig. 7). The SMA of Variance was the lowest index in each axis; thus, it was difficult to detect differences in CB and OB. However, all axes of SMA of Range*–XYZ* showed obvious difference, as shown by the values in Table 3.

**Table 3:** Inertial sensor results

	OB	CB	OO	CO
MALA–*X*	0.44 ± 0.20	0.36 ± 0.38	1.24 ± 0.78	3.05 ± 1.48
MALA–*Y*	0.56 ± 0.11	0.55 ± 0.11	1.42 ± 1.23	3.05 ± 2.03
MALA–*Z*	0.74 ± 0.21	0.68 ± 0.22	1.26 ± 0.81	2.65 ± 1.51
MALA–*XYZ*	1.75 ± 1.11	1.62 ± 1.05	3.937 ± 2.48	8.75 ± 4.89
RMS–*X*	0.59 ± 0.22	0.53 ± 0.21	1.92 ± 1.80	4.88 ± 2.43
RMS–*Y*	0.70 ± 1.4	0.71 ± 1.03	2.21 ± 2.05	5.42 ± 3.52
RMS–*Z*	0.94 ± 0.20	0.88 ± 0.21	1.89 ± 1.69	4.72 ± 2.56
RMS–*XYZ*	1.47 ± 0.86	1.39 ± 0.871	3.57 ± 3.16	8.55 ± 4.74
MAD–*X*	− 0.59 ± 0.59	−0.43 ± 0.39	−1.08 ± 0.62	−3.00 ± 1.86
MAD–*Y*	−0.95 ± 2.19	−0.88 ± 2.18	−1.83 ± 2.35	−3.14 ± 2.26
MAD–*Z*	−0.59 ± 0.59	−0.87 ± 0.75	−1.38 ± 1.59	−2.54 ± 2.06
MAD–*XYZ*	−2.12 ± 1.97	−2.15 ± 2.05	−4.32 ± 3.24	−8.69 ± 5.57
SMA of Range–*X*	1.42 ± 0.53	1.31 ± 0.44	7.33 ± 7.40	18.21 ± 9.62
SMA of Range–*Y*	1.41 ± 1.11	1.44 ± 1.04	3.59 ± 6.60	21.80 ± 14.63
SMA of Range–*Z*	1.88 ± 0.41	1.85 ± 0.30	5.91 ± 4.47	15.88 ± 11.42
SMA of Range–*XYZ*	2.57 ± 0.96	6.30 ± 9.88	12.89 ± 10.86	35.23 ± 22.70
SMA of Variance–*X*	0.0013 ± 0.0010	0.0013 ± 0.0011	0.39 ± 1.41	0.18 ± 1.631
SMA of Variance–*Y*	0.0010 ± 0.0007	0.0010 ± 0.0006	0.06 ± 0.15	0.29 ± 0.32
SMA of Variance–*Z*	0.0028 ± 0.0010	0.0026 ± 0.0008	0.04 ± 0.11	0.15 ± 0.17
SMA of Variance–*XYZ*	0.0013 ± 0.0010	0.0013 ± 0.0011	0.27 ± 0.76	1.15 ± 1.05
ZCR–*X*	0.31 ± 0.28	0.29 ± 0.29	0.46 ± 0.17	0.48 ± 0.12
ZCR–*Y*	0.24 ± 0.27	0.23 ± 0.21	0.4 ± 0.16	0.45 ± 0.07
ZCR–*Z*	0.45 ± 0.31	0.29 ± 0.24	0.47 ± 0.24	0.52 ± 0.17
CBA–*XY*	0.12 ± 0.16	0.17 ± 0.23	0.24 ± 0.11	0.42 ± 0.18
CBA–*XZ*	0.43 ± 0.28	0.43 ± 0.28	0.24 ± 0.13	0.51 ± 0.14
CBA–*YZ*	0.16 ± 0.15	0.14 ± 0.16	0.22 ± 0.12	0.35 ± 0.19

All units: 1/100 g = 9.8 m/s^2^; ZCR, CBA unit: 100%

The Wilcoxon signed-rank test results are presented in Table 4. No differences were found in any of the 25 indexes but were found for the index of SMA of Range–*XYZ* when subjects stood on both feet (OB–CB). In cases OB–OO and OB–CO, there were differences in most indexes (both 24/26). Similarly, only CBA values were not recognized for the CB–OO case. Furthermore, significant differences were found in most indexes for cases CB–CO and OO–CO (25/26 and 22/26, respectively).

**Table 4:** *p* values of inertial sensor for static posture

	OB–CB	OB–OO	OB–CO	CB–OO	CB–CO	OO–CO
MALA–*X*	0.334	0.001*	0.001*	0.001*	0.001*	0.003*
MALA–*Y*	0.532	0.001*	0.005*	0.001*	0.005*	0.027*
MALA–*Z*	0.363	0.005*	0.001*	0.003*	0.001*	0.005*
MALA–*XYZ*	0.334	0.001*	0.001*	0.001*	0.001*	0.008*
RMS–*X*	0.281	0.001*	0.001*	0.001*	0.001*	0.006*
RMS–*Y*	0.82	0.001*	0.002*	0.001*	0.002*	0.017
RMS–*Z*	0.307	0.001*	0.001*	0.001*	0.002*	0.008*
RMS–*XYZ*	0.256	0.001*	0.001*	0.001*	0.001*	0.011*
MAD–*X*	0.334	0.028*	0.001*	0.001*	0.001*	0.003*
MAD–*Y*	0.126	0.001*	0.011*	0.001*	0.009*	0.053
MAD–*Z*	0.069	0.027*	0.002*	0.233	0.005*	0.009*
MAD–*XYZ*	0.712	0.001*	0.003*	0.001*	0.003*	0.023*
SMA of Range–*X*	0.363	0.001*	0.001*	0.001*	0.001*	0.008*
SMA of Range–*Y*	0.589	0.001*	0.001*	0.001*	0.001*	0.005*
SMA of Range–*Z*	0.755	0.001*	0.001*	0.001*	0.001*	0.005*
SMA of Range–*XYZ*	0.012*	0.001*	0.001*	0.047*	0.001*	0.005*
SMA of Variance–*X*	0.789	0.001*	0.001*	0.001*	0.001*	0.012*
SMA of Variance–*Y*	0.645	0.001*	0.001*	0.001*	0.001*	0.006*
SMA of Variance–*Z*	0.527	0.001*	0.001*	0.001*	0.001*	0.009*
SMA of Variance–*XYZ*	0.288	0.001*	0.001*	0.001*	0.001*	0.009*
ZCR–*X*	0.865	0.065	0.029*	0.041*	0.017*	0.221
ZCR–*Y*	0.925	0.039*	0.025*	0.002*	0.005*	0.125*
ZCR–*Z*	0.105	1.000	0.513	0.01*	0.003*	0.181
CBA–*XY*	0.414	0.017*	0.003*	0.149	0.013*	0.009*
CBA–*XZ*	1.001	0.025*	0.256	0.069	0.394	0.001*
CBA–*YZ*	0.239	0.099	0.02*	0.083	0.013*	0.023*

*Significance, *p* < 0.05

The initial result thus indicates that the sensors performed the best for the CB cases (i.e., OB–CB, CB–OO, and CB–CO) when compared with other actions. The OB–CB case showed no differences other than the SMA of Range–*XYZ*; however, there were significant differences in the CB–OO and CB–CO cases. Overall, the results indicate that the sensor has difficulty distinguishing whether a subject’s eyes are opened or closed but can distinguish whether they are standing on one foot or two.

When compared to other features, ZCR and CBA did not identify differing results for a variety of static balance measurement actions and could not recognize the differences between situations.

### Results of multiscale entropy analysis

**Fig. 8:**
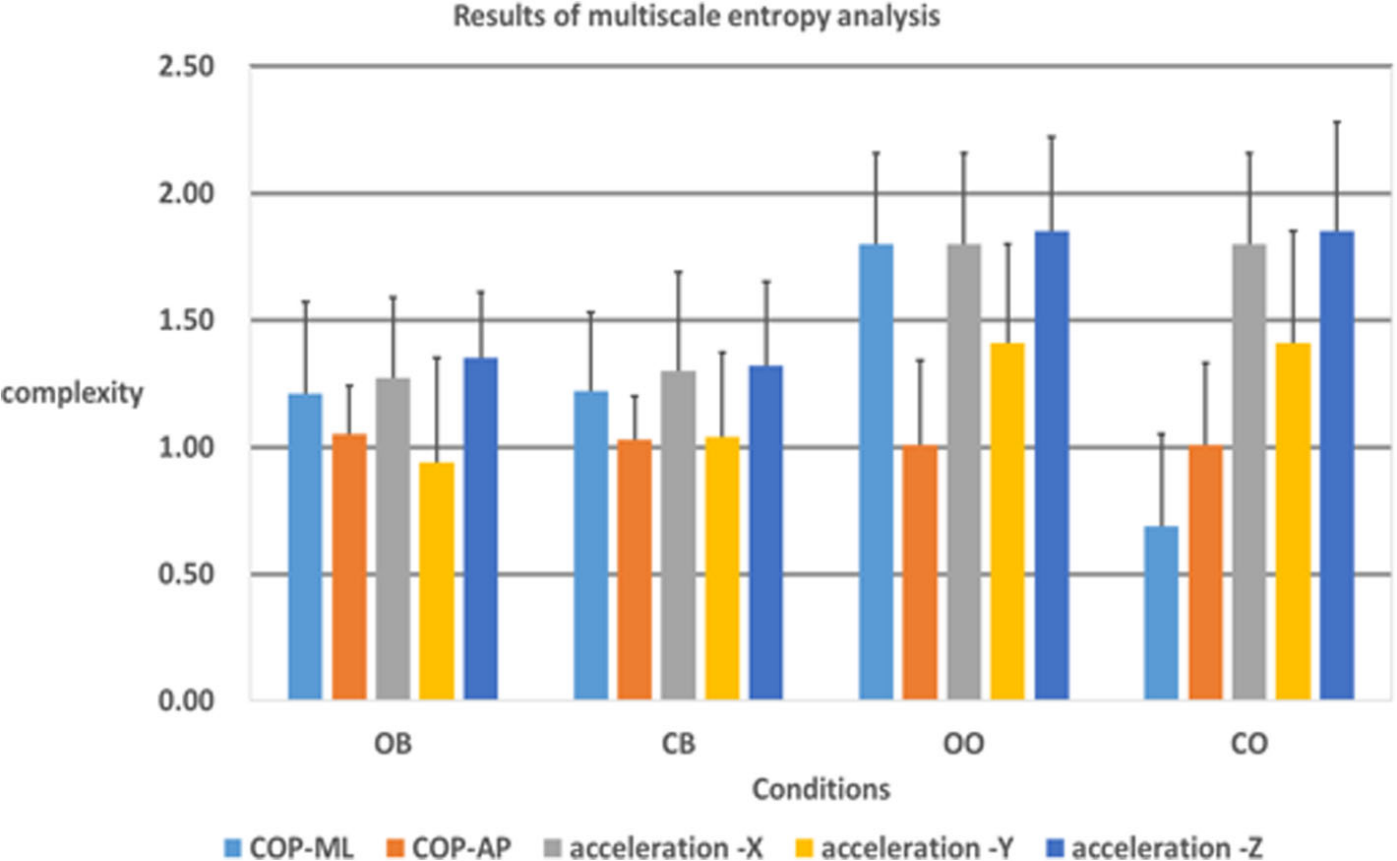
Results of multiscale entropy analysis

MSE can be used to quantify complexity in widely varying timescales. Whether greater MSE values mean higher complexity, greater physiological complexity indicates a greater degree of adaptability to the external environment or vice versa. Results of the MSE analysis of COP and acceleration are presented in Table 5. As complexity becomes higher, adaptability becomes better. Table 7 shows that all ML-AP conditions with COP showed significant differences. In addition, Fig. 8 shows that condition OO (open eyes and standing on one foot) has the highest complexity. Therefore, as shown in Table 5, condition OO shows higher complexity at the *X*, *Y*, and *Z* axes for acceleration than the other conditions, whereas the force plate results showed that the ML axis is higher for COP than for others.

**Table 5:** Results of multiscale entropy analysis

	OB	CB	OO	CO
COP-ML	1.21 ± 0.36	1.22 ± 0.31	1.80 ± 0.36	0.69 ± 0.36
COP-AP	1.05 ± 0.19	1.03 ± 0.17	1.01 ± 0.33	0.83 ± 0.32
Acceleration-*X*	1.27 ± 0.32	1.3 ± 0.39	1.8 ± 0.36	1.57 ± 0.36
Acceleration-*Y*	0.94 ± 0.41	1.04 ± 0.33	1.41 ± 0.39	1.29 ± 0.44
Acceleration-*Z*	1.35 ± 0.26	1.32 ± 0.33	1.85 ± 0.37	1.56 ± 0.43

A Wilcoxon signed-rank test analysis was also performed on the MSE results; results for each measurement tool condition are presented in Table 6. Almost all experimental actions were identifiable by AP COP data, whereas ML data could only be used to identify OB–CO and CB–CO. Only OB–OO, OB–CO, and CB–OO were distinguishable in the *Y*-axis using an inertial sensor as the balance measurement tool, whereas in the *X* and *Z* axes, OB–OO and CB–OO could be distinguished. In the *Z*-axis, OO–CO was distinguishable. Thus, MSE could not be used to discern OB–CB regardless of whether a force plate or acceleration sensor was used. For the preliminary OO and cases OB–OO, CB–OO, and OO–CO, MSE could be used to identify differences between standing on one foot and standing on two feet.

**Table 6:** Identification of four experimental actions with axial data

	OB–CB	OB–OO	OB–CO	CB–OO	CB–CO	OO–CO
ML	0.488	0.932	0.008*	0.629	0.011*	0.074
AP	0.82	0.012*	0.006*	0.009*	0.005*	0.035*
*X*	0.91	0.002*	0.078	0.006*	0.125	0.083
*Y*	0.116	0.008*	0.023*	0.012*	0.125	0.307
*Z*	0.572	0.002*	0.164	0.002*	0.233	0.047*

*Significance, *p* < 0.05

Different experimental actions were distinguishable by all different axial data from the force plate, whereas the inertial sensor could not be used to distinguish the *X*–*Z* axial data, as summarized in Table 7.

**Table 7:** Identifying force plate and inertial sensor axial data from experimental actions

	COP	Acceleration		
	ML-AP	*X*–*Y*	*X*–*Z*	*Y*–*Z*
OB	0.017*	0.030*	0.594	0.005*
CB	0.023*	0.118	0.975	0.099
OO	0.001*	0.003*	0.320	0.001*
CO	0.001*	0.003*	0.712	0.005*

*Significance, *p* < 0.05

## Discussion

The force plate and inertial sensor measurements indicated which index and feature had the most results with similar trends. A cursory observation of the index scores of force plate and acceleration showed that the smaller values were better. Force plate measurements indicated that subjects were most stable under the condition of OB, whereas inertial sensor measurement features indicated they were most stable under CB. In our study, we found that better results were obtained without visual aid. The reason for this difference from our expectation is because Cooper et al. [44] reported in a test involving standing on one leg with eyes closed that men and women who could hold the position for < 2 s were thrice more likely to die than those who could hold the position for ≥10 s; however, individuals who could not perform the test were around 12 times more likely to die in the following 13 years. They concluded that the underlying message is that “even a little helps – at least as far as physical activity is concerned.” We obtained a conclusion that closing eyes can enable subjects to understand their own physical status and predict risk. In addition, some subjects in our study may have been subhealthy, a characteristic that cannot be visually observed, resulting in differences from what we expected. This difference also indicates that equipment types can influence experimental results. This also shows that if an experimental task contains visual feedback in static balance, then COP data can distinguish the difference. This is mainly because to maintain postural stability, humans will control the stability of both their feet so that their body stays on the underlying surface [6]. Song et al. [8] found from COP that visual feedback can aid subjects in adjusting their body posture to maintain the body balance, which is explained in Table 2 wherein in conditions OB–CO and CB–CO show differences for all indexes, indicating that it is difficult to maintain self-balance based on proprioception when input from the visual system is lost. Thus, if a task contains visual feedback in static balance, COP data can be used to distinguish the difference. As one grows older and proprioception weakens, vision becomes more important for balance control [45, 46]. Thus, it is suggested that the elderly used a force plate to measure static balance because of the visual system. Otherwise, based on each index’s results, MDIST, MDIST-AP, RDIST, RDIST-AP, and 95% CC presented differences in each situation. This may be because average calculation indexes more easily distinguish differences in chosen indexes.

Inertial sensor indexes presented results that were slightly different from those of the force plate. Case OB–CB showed no difference other than the SMA of Range–*XYZ*, but showed significant differences for cases CB–OO and CB–CO. Furthermore, accelerometer data were indistinguishable between the open and closed eyes (OB–CB) tests but could be used to determine whether the subject was standing on one foot or both feet (OB–OO). Thus, this indicates that while it is easier to measure lower limbs using a sensor, the sensor is less likely to be affected by the visual system. This result agrees with findings from previous studies wherein a sensor was strapped to the lower back, including the pelvis, sacrum, and the L3 to L5 vertebrae [11, 47, 48]. The accelerometer is generally worn on the waist because the person moves as a whole during physical activity and the waist is closer to the body’s center of gravity. Thus, the detected value is closer to the actual amount of physical activity [14, 42, 49]. When investigating postural stability based on the visual system, a force plate is often used for data collection because a subject controls their stability with both feet to maintain balance [45]. Song et al. [8] found from COP data that visual feedback helps subjects adjust posture to maintain balance.

There are no clear results for ZCR, probably because the original application of the zero-crossing rate is the regular motion of measuring the number of steps of walking. However, in this experiment, the irregular motion is not good as the characteristic value. All four experiment actions had very low CBA correlation coefficients and a very large standard deviation, indicating that the data was insufficiently accurate. Further, ZCR and CBA could not be used to distinguish between the OB–OO and CB–OO cases. These results were not as expected as these indexes were thought to be suitable for measuring static balance [50]. We infer that the original eigenvalue was derived from that of the gait experiment and belongs to the motion law experimental type. These facts may be responsible for this feature’s poor results.

The statistical Wilcoxon signed-rank test with experimental actions, force plate data, and inertial sensor axial data are used for calculating MSE. In comparison with the balance data obtained by the inertial sensor, the MSE data obtained by the force plate allowed us to distinguish the actions studied more easily. When using the force plate as a balance measurement tool, almost all experimental actions could be distinguished in the forward–backward direction. Inertial sensors have less commonality, but there were no differences in measurements between actions with or without vision. The force plate allowed for easy distinguishing between two axes for OO. Although it is possible to distinguish actions in the *YZ* axial with the inertial sensor, there is no experimental action that allows for distinguishing the *XZ* axes. However, both *YZ* and *XA* axes can be distinguished with the force plate. In addition, previous research [21, 43] demonstrates that trials in which subjects stand on two legs with their eyes open exhibit the best performance, with features similar to those of force plate measurements performed herein. However, this was not the case for most sensor-related features. Thus, a force plate is recommended to evaluate postural stability based on the results of static equilibrium force measurements.

Using MSE as a common feature for examining the findings showed that the results obtained with the force plate, inertial sensor, and MSE are not mutually consistent. The force plate results show that the best postural stability was achieved under the OB condition, whereas the inertial sensor results show that the best postural stability was achieved under the CB condition. The MSE results indicated that the sensor of acceleration *X*, *Y*, *Z* lead to the best adaptability under the OO condition as well as the best adaptability under the force plate-ML of the OB condition. Based on the results presented in Table 6, the MSE results were significantly different in the cases containing OO (OB–OO, CB–OO, and OO–CO). Thus, MSE can be used to distinguish different actions. The complexity of MSE can be used to understand the adaptability of postural stability [51], especially the AP of the force plate, and the *Z*-axis (forward/backward) of the inertial sensor. These results indicate that standing on one foot with eyes open shows better results than standing on two feet with both eyes open. These results can be explained in two parts. Sun and Lee [17] found that in the static balance for postural stability challenge exergame, subjects felt that the dynamic humanoid frame (standing on one foot with eyes open) is easier than the static humanoid frame (standing on both feet with eyes open) because it is easier to maintain postural stability. In addition, their study found that when subjects stood on both feet with eyes open, their postural stability control was better when the time duration was shorter. In our study, we have tried to discuss the different measurements of postural stability. The respective durations for the four actions designed in our study were 30 s each; all our subjects were healthy and young. The results of the balance tests conducted with visual assistance were non-challenging. The results correspond with those of Cooper et al. [44], who reported that closing the eyes can enable subjects to understand their physical status, improve risk prediction, and let young subjects predict signs of early warning in terms of any difficult situations. On the other hand, we may be able to reduce the time for further evaluation of warning signs in the experiment or in the community services. Otherwise, considering that we hypothesize Cooper et al.’s [44] findings, it is suggested that the use of a combination of physical performance measurements increases prognostic power in the analyses of middle-aged populations. We can further discuss the assessment of different measurements of postural stability and whether the same associations are present.

Under static conditions, the force plate was found to be more sensitive to actions influenced by the visual system. In contrast, under acceleration, the inertial sensor was more sensitive to experimental actions that were influenced by the lower limbs, as was found by Keshner et al. [3]. Thus, when the visual environment changes while standing still, the head and torso (sensor) movements of the subject will be greater than those of the lower extremities. To maintain balance, the subject controls the double-foot stability to maintain their lower limbs, but the head and torso posture is still be affected by visual changes as shown by subjects taking normal strides while walking during the test.

## Conclusions

This study aimed to (1) investigate different equipment types for balance testing in healthy subjects and (2) explore the conditions that would affect subjects’ postural stability using multiscale complexity analysis. It was anticipated that our findings would suggest performing investigations on different evaluation tool choices that can be easily adapted to suit different needs. The results for the complexity index and traditional balance indicators are comparable in their implications on different conditions. The experimental findings suggest that it is better to use a force plate if the task is based on the visual system, whereas an inertial sensor should be used for lower limb tasks. From the MSE results, we can see that both force plate and accelerations showed condition OO (open eyes and standing on one foot) as the best condition, from which axial directions can be identified. Our study is different from previous studies, in that previous studies have used MSE to determine the physiological and pathological events of aging [11, 12, 18–21, 51, 52], whereas we used MSE to determine the equipment that measures the performance of postural stability. We attempted to generalize the applications of complexity index to tasks [53] and training characteristics and to explore different tools to obtain different results.

## Abbreviations

AP: Anterior-posterior
CB: Closed eyes, standing on both feet
CBA: Correlation between axes
CI: Complexity index
CO: Closed eyes, standing on one foot
COP: Center-of-pressure
ML: Medio-lateral
OB: Open eyes, standing on both feet
OO: Open eyes, standing on one foot
RMS: Root mean square
SMA: Simple moving average
TOTEX: Total excursions
ZCR: Zero cross rate
